# Exploring Communication Tools in Clinical Settings: Addressing the Need for a Universal Approach to Clinical Handover

**DOI:** 10.1155/nrp/2713238

**Published:** 2026-01-15

**Authors:** Alice Mei Ling Chan, Wai Kwong Poon, Belle Yuen Ching Lau, Rick Yiu Cho Kwan

**Affiliations:** ^1^ School of Nursing, Tung Wah College, Hong Kong, China, twc.edu.hk; ^2^ School of Arts and Humanities, Tung Wah College, Hong Kong, China, twc.edu.hk

**Keywords:** clinical handovers, healthcare professionals training, structured communication frameworks

## Abstract

**Background:**

Effective communication in clinical handovers is critical for promoting patient safety and care quality. Healthcare professionals lack interprofessional training on clinical handovers.

**Aims:**

To identify educational strategies used to enhance clinical handover competence in clinical settings, map outcomes used to evaluate training effectiveness, and explore methods suitable for interprofessional education.

**Design:**

A scoping review guided by the PRISMA‐ScR checklist.

**Methods:**

A systematic search of seven electronic databases was conducted.

**Results:**

Seventeen studies were included in this review, which yielded the following major themes: (1) education strategies were evidence‐based program structured with communication frameworks; (2) diverse outcomes were measured in clinical handover training; (3) inadequacy in interprofessional training on clinical handovers. Training programs encompassed various learning activities to improve clinical handover practices. Few studies highlight effective training methods enhanced communication, confidence, and operational efficiency.

**Conclusions:**

There is a need for more robust, longitudinal, and randomized studies to evaluate long‐term effects. Future research should expand interprofessional training, incorporate innovative technologies, and develop standardized curricula to better prepare healthcare professionals for effective clinical handovers. Results indicate that no single tool has been universally recommended. There remains a need to recommend a universal handover tool through interprofessional education and promote its use within diverse healthcare disciplines.

## 1. Introduction

Over the past decade, a range of strategies have been introduced to enhance staff’s competency on patient safety, including the crucial attitudes, knowledge, and skills that enable healthcare providers to reduce avoidable risks and harms to patients [1, 2]. Numerous patients suffer harm while hospitalized because of errors and adverse events [3], ineffective communication during handovers, and inadequate incorporation of patient safety education into the curricula of health professional training institutions [2].

Clinical handover is the communication process through which healthcare professionals exchange patient information among teams [4]. Research showed that ineffective handovers are linked to various hazards including missing arrangement of required equipment for patients, missing information, diagnosis errors, and delayed treatment [5]. The World Health Organization stresses the significance of teamwork and clear communication at every healthcare level to enhance interprofessional collaboration and communication, thus preventing health‐related errors [6]. Patients receive ongoing care and services from a range of medical professionals in the hospital. Effective communication among staff members is crucial to ensure that they are being informed about the patients’ progress and change if any. Doctors, nurses, and allied health professionals need training to guarantee effective communication during clinical handover process. Clinical handovers can be commonly trained through simulation [7] or real‐life experiences during clinical placement [8–10]. Simulation necessitates the presence of a facilitator and equipment setup, while clinical practice is hindered by heavy workloads and a lack of supervision. Consequently, both approaches pose challenges to the training success in clinical handovers.

A review conducted by Palese et al. [11] revealed that nursing students had limited exposure to clinical handover practices. This study showed that doctors faced challenges in clinical handovers because of insufficient educational and learning opportunities [12]. There is a lack of comprehensive training on clinical handovers, with only specific communication tools such as situation, background, assessment, and recommendation (SBAR) being introduced within communication skills courses, albeit without a concentrated emphasis on its effective application for clinical handovers. A review done by Desmedt et al. [5] concluded that establishing a shared understanding among disciplines is crucial for enhancing interdisciplinary communication, both verbally and in writing, particularly when transferring a patient’s information across disciplines. Numerous studies have delved into interprofessional education, with a particular emphasis on evaluating staff preparedness, attitudes, and patient care delivery like case management. However, there is a scarcity of research promoting interprofessional clinical handover training and assessing effectiveness. Desmedt et al. [5] also suggested implementing curricular interventions tailored for diverse audiences, given that handover is a diverse activity that demands a multidisciplinary approach. Promoting interprofessional education is considered vital to flatten hierarchies and improve teamwork during handovers, supporting better patient safety culture.

### 1.1. Aims

The objectives of this scoping review are as follows:

1. To identify the training methods and communication frameworks employed to enhance clinical handovers in healthcare settings;
2. To map out the measured outcomes used to evaluate the effectiveness of training programs designed to improve clinical handovers in healthcare settings; and
3. To explore training methods suitable for interprofessional education in the enhancement of clinical handovers.

## 2. Methods

To examine the current educational strategies on improving clinical handover in clinical settings, a scoping review is considered to be a suitable methodology. The general purpose of a scoping review is to identify and map those available evidence [13, 14] and ascertain the breadth of literature available, offering a comprehensive overview of the volume of studies accessible and providing a broad summary of its focus on the topic concerned [15].

### 2.1. Design

The scoping review guidelines from Arksey and O’Malley [14] and Joanna Briggs Institute [16] were used to guide this review. The framework has five stages: (1) develop the research question; (2) identify relevant studies; (3) select eligible studies; (4) extract and chart the data; and (5) compile, summarize, and present the results. We reported our study according to the Preferred Reporting Items for Systematic reviews and Meta‐Analyses extension for Scoping Reviews (PRISMA‐ScR) guidelines [17, 18]. This guideline enhanced the review process with clarity, structure, and rigor (Supporting File 1).

### 2.2. Methods and Search Strategy

#### 2.2.1. Stage 1: Develop Research Questions

The initial stage included a preliminary searching of literature to identify knowledge gaps on education strategies to promote clinical handover competence in clinical settings. The following research questions were formulated: (1) What are the training methods and communication frameworks employed to enhance clinical handovers in clinical settings? (2) What are measured outcomes used to evaluate the effectiveness of training programs designed to improve clinical handovers in clinical settings? and (3) What are those training methods suitable for interprofessional education to enhance clinical handovers?

#### 2.2.2. Stage 2: Identify Relevant Studies and Search Strategy

Search strategy was developed based on the population, concept, and context (PCC) framework as demonstrated in Table 1. The PCC framework for this study consisted of the following: population being the “healthcare professionals”; the concept being the “trainings or interventions”; and the context being the “settings where clinical handover occurs.”

**Table 1:** PCC characteristics of the study review question.

Population	Healthcare professionals encompass licensed individuals, including doctors, nurses, physical therapists, and occupational therapists
Concept	Trainings and interventions in promoting clinical handover such as simulation, clinical practice, and tutorial
Context	Settings where clinical handovers occur such as clinical practicum in clinical environment, training in simulation laboratory, and learning activities in classroom

#### 2.2.3. Stage 3: Select Eligible Studies

Peer‐reviewed original literature retrieved from the databases was considered to be eligible.

##### 2.2.3.1. Eligibility Criteria

The inclusion and exclusion criteria were applied to determine studies’ eligibilities. The inclusion criteria were taken as follows: (1) studies focused on trainings or interventions to improve clinical handover in clinical settings; (2) studies had been peer‐reviewed; (3) studies with primary findings; (4) studies were reported in English; and (5) studies were published between 2015 and 2025.

The exclusion criteria were those studies which were: (1) not involving trainings or interventions; (2) not focusing on clinical handover; and (3) not having any primary findings such as discussion papers or reviews.

##### 2.2.3.2. Selection of Studies for Inclusion

Literature searches on electronic databases were conducted on 16th–25th December 2024. The first author (A.M.L.C.) performed the literature search in consultation with a librarian and supervisor (R.Y.C.K.) and the search was conducted in the following databases: PubMed, ERIC, Journals@Ovid, PsycINFO, CINAHL, MEDLINE Ultimate, and Academic Search Ultimate. The combination of Medical Subject Headings (MeSH) with Boolean terms AND/OR applied to combine search strings. The following keywords were used in the searches: {(healthcare OR health care) AND (training OR education) AND (clinical handover OR shift handover OR handoff)}, and it was entered in PubMed for searching the literature. The co‐author (W.K.P.) independently verified search terms and discussed initial search results to confirm the searching strategy.

#### 2.2.4. Stage 4: Extract and Chart the Data

The reviewer (A.M.L.C.) created a data‐charting form based on the research questions using a Microsoft Excel spreadsheet, which was then commented by the supervisor (R.Y.C.K.), and the form was revised. Studies meeting the eligibility criteria were extracted, including data on the authors, year of publication, country of origin, research design, study setting, healthcare professionals, number of recruited participants, training methods and frameworks, and measured outcomes. Each reviewer (A.M.L.C., W.K.P., B.Y.C.L.) independently read and filled in the form. Initial results were discussed to achieve consensus before proceeding with the iterative process of updating the data‐charting form.

#### 2.2.5. Stage 5: Compile, Synthesize, and Present the Results

The identified studies were thematically synthesized based on review questions concerning the training intervention, communication framework, measured outcomes, and healthcare participants, following a three‐stage process: (1) the data were coded line by line in a free‐form manner; (2) these “free codes” were then grouped into relevant categories to share “descriptive” themes; and (3) the themes further formulate into “analytical” themes [19, 20]. Following Arksey and O’Malley [14], no quality appraisal of the included studies was conducted.

## 3. Results

**Figure 1:**
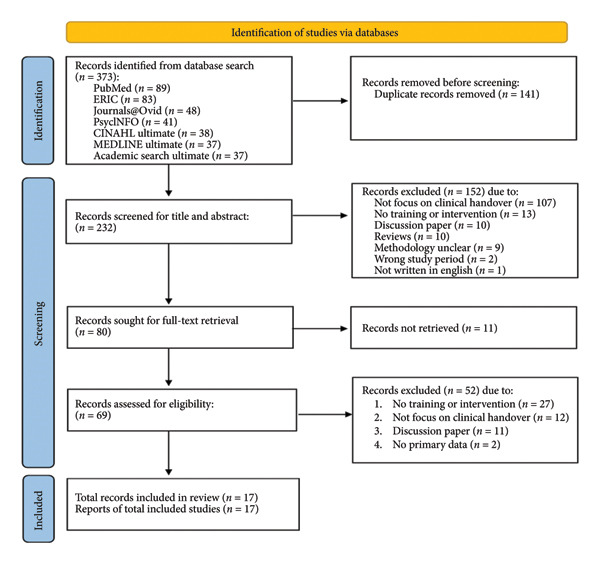
Flow diagram of the study selection process.

We grouped the studies by populations and study designs, along with the education strategies, communication frameworks, and findings from quantitative and qualitative perspectives. See flow diagram of the study selection process (Figure 1) for a detailed overview of the study selection.

### 3.1. Screening Results

The search for electronic databases resulted in 373 articles. The Rayyan system was utilized for screening purposes. A total of 373 studies were imported for screening; after removing 141 duplicates, 232 studies were left for title and abstract screening. Pilot screening was carried out to ensure consistency among reviewers. Each reviewer (A.M.L.C., W.K.P., B.Y.C.L.) independently assessed 10 randomly selected titles and abstracts for alignment. The consensus was reached after pilot screening with 152 studies deemed irrelevant, leaving 80 studies for full‐text screening. Eleven records that were not retrieved resulted in 69 studies remaining for eligibility assessment. Fifty‐two records were subsequently excluded, with 17 studies meeting the eligibility criteria. All screening processes were independently carried out by three reviewers (A.M.L.C., W.K.P., B.Y.C.L.). Five studies were retrieved for a pilot data extraction exercise.

### 3.2. Studies Characteristics

As shown in Table 2, 17 studies were retained for further reviewing. Data for the studies were collected in the United States (*n* = 8); United Kingdom (*n* = 2); Sudan (*n* = 1); Australia (*n* = 1); Palestine (*n* = 1); Argentine Republic (*n* = 1); Norway (*n* = 1); Korea (*n* = 1); and Brazil (*n* = 1). Regarding study designs, the 17 studies included were mixed‐method studies (*n* = 3), qualitative studies (*n* = 1), quasi‐experimental (*n* = 12), and randomized controlled trials (*n* = 1).

**Table 2:** Characteristics of the included studies.

Studies	Year	Country	Study design				Settings		Healthcare professionals					
			Mixed‐method	Qualitative	Quasi‐experimental	Randomized control trial	Clinical	Academic	Doctor	Nurse	Allied health	Trainee/student	Interprofessional	Participants numbers *N* =
1. Acharya et al.	2017	UK	√					√	√			√		11
2. Adam et al.	2022	Sudan			√		√		√					48
3. Beament et al.	2018	Australia	√				√		√	√	√		√	29
4. Cunningham et al.	2021	USA	√					√	√		√	√	√	66
5. Fahajan et al.	2023	Palestine			√		√			√				51
6. García Roig et al.	2020	Argentine Republic			√		√		√					NA
7. Haddeland et al.	2022	Norway		√				√	√	√			√	17
8. Holt et al.	2020	UK			√			√	√			√		41
9. Lebensohn‐Chialvo et al.	2022	USA			√		√			√	√	√	√	26
10. Lim & Kang	2024	Korea			√			√		√				34
11. Reilly et al.	2024	USA			√		√		√					NA
12. Ronnebaum et al	2023	USA			√			√			√	√		52
13. Soares et al.	2023	Brazil			√		√		√					23
14. Stuart et al.	2021	USA				√		√		√		√		69
15. Usher et al	2018	USA			√		√			√				32
16. Vanderzwan et al	2023	USA			√			√		√		√		56
17. Wollenhaup et al	2017	USA			√		√			√				28

Regarding participants in the studies, 15 studies included participant details with an average of 38.87 participants (min = 11, max = 69, SD = 16.34). In two of the studies, participant details were not provided; instead, one measured the number of assessments conducted [21], and the other measured the number of observations [22]. The studies involved different healthcare professionals, doctors (*n* = 9), nurses (*n* = 9), allied health professionals (*n* = 4), and trainees or students (*n* = 7), and there were only four studies that involved interprofessional training. The training occurred in clinical settings (*n* = 9) and academic settings (*n* = 8).

### 3.3. Study Findings

Finally, three themes were identified: (1) education strategies were evidence‐based program structured with communication frameworks; (2) diverse outcomes were measured in clinical handover training; (3) inadequacy in interprofessional training on clinical handovers.

#### 3.3.1. Theme 1: Education Strategies Were Evidence‐Based Program Structured With Communication Frameworks

As shown in Table 3, the training methods examined in the included studies could be classified into two primary groups: conventional methods and simulations. Conventional methods such as lectures with PowerPoint presentations on handover (*n* = 9), videos illustrating handover of variable quality (*n* = 8), small group discussions (*n* = 6), debriefing sessions (*n* = 4), workshops (*n* = 3), curriculum‐based (*n* = 2), and specialized training course (*n* = 1) were utilized. Simulation‐based training programs involved the utilization of simulators (*n* = 4), standardized patients (*n* = 3), role‐playing scenarios (*n* = 3), and virtual simulations (*n* = 2). There wasn’t just one activity in the training program; rather, most of the trainings incorporate multiple methods. In addition to face‐to‐face learning, online learning was utilized for lectures (*n* = 2), video telehealth (*n* = 1), debriefing (*n* = 1), and simulation (*n* = 1).

**Table 3:** Training methods and communication frameworks.

Studies	Conventional							Simulation				Framework					
	Lecture	Video	Discussion	Debrief	Workshop	Curricular	Training course	Sim^a^	Standardized patient	Roleplay	Virtual Sim	SBAR^b^/ISBAR^c^/iSoBAR^d^/SBART^e^	I‐PASS^f^	TeamSTEPPS^g^	PASS‐BAR^h^	SAFETIPS^i^	N‐PAS^j^
1. Acharya et al., 2017	√	√	√					√				√					
2. Adam et al., 2022			√									√					
3. Beament et al., 2018	√	√										√					
4. Cunningham et al., 2021				√		√			√			√	√	√			
5. Fahajan et al., 2023							√					√					
6. García Roig et al., 2020		√			√					√			√				
7. Haddeland et al., 2022	√							√				√					
8. Holt et al., 2020	√	√	√		√					√		√					
9. Lebensohn‐Chialvo et al., 2022		Tele health		Video					√			√					
10. Lim & Kang, 2024	√		√	√				√		√					√		
11. Reilly et al., 2024	√					√										√	
12. Ronnebaum et al, 2023				F2F^k^ & online					√			√		√			
13. Soares et al., 2023	Online	√			√			Online					√				
14. Stuart et al, 2021		√									√	√					
15. Usher et al, 2018	Online	√										√					
16. Vanderzwan et al, 2023	√		√								√						√
17. Wollenhaup et al, 2017			√									√					

^a^Sim = simulation.

^b^SBAR = “Situation, Background, Assessment, Recommendation”.

^c^ISBAR = “Identify, Situation, Background, Assessment, Recommendation”.

^d^iSoBAR = “Identify, Situation, Observation, Background, Assessment/Action, Read‐back/Responsibility”.

^e^SBART = “Situation, Background, Assessment, Recommendation, Thank”.

^f^I‐PASS = “Illness severity, Patient summary, Action items, Situation awareness, and contingency planning, Synthesis by receiver”.

^g^TeamSTEPPS = “Team Strategies & Tools to Enhance Performance & Patient Safety”.

^h^PASS‐BAR = “Patient, Assessment, Situation, Safety concerns, Background, Action, Rcommendation”.

^i^SAFETIPS = “Stats, Assessment, Focused plan, Exam, To do, If/then, Pointers/pitfalls, Sick‐o‐meter”.

^j^N‐PAS = “Nursing, Patient summary, Action plan, Synthesis”.

^k^F2F = face‐to‐face.

Communication frameworks were commonly used in the training programs, they were SBAR and its derivatives (*n* = 12), I‐PASS (Illness severity, Patient summary, Action list, Situation awareness and contingency planning, Synthesis by receiver) (*n* = 3), TeamSTEPPS (Team Strategies & Tools to Enhance Performance & Patient Safety) (*n* = 2), PASS‐BAR (Patient, Assessment, Situation, Safety concerns, Background, Action, Recommendation) (*n* = 1), SAFETIPS (Stats, Assessment, Focused plan, Exam, To do, If/then, Pointers/pitfalls, Sick‐o‐meter) (*n* = 1), and N‐PAS (Nursing, Patient summary, Action plan, Synthesis) (*n* = 1). SBAR and its derivatives were frequently utilized by various healthcare professionals during their individual professional training or interprofessional training sessions. I‐PASS was explicitly tailored for doctors in clinical practice, resulting in its exclusive use by this professional group, but with limited adoption by others. While doctors and allied health professionals incorporated TeamSTEPPS into their practices, nurses did not engage with this particular framework. Only two studies, which involved nurses, utilized PASS‐BAR and N‐PAS for training purposes.

#### 3.3.2. Theme 2: Diverse Outcomes Measured in Clinical Handover Training

A wide array of measurement tools were utilized across these 17 studies, encompassing surveys [12, 23–25], communication framework sheets [21, 23, 26–29], knowledge assessment questionnaires [25, 27, 29–31], safety attitudes questionnaire [30], handover evaluations [22, 25, 28, 32–34], learning feedback forms [32, 34, 35], observational checklists [35], and perceived self‐efficacy assessments [32].

As shown in Table 4, measured outcomes were diverse including communication (*n* = 6), information completeness and documentation (*n* = 6), attitudes toward patient safety (*n* = 3), participants’ competence (*n* = 2), knowledge (*n* = 2), reduction in handover interruptions and time (*n* = 2), participants’ confidence and self‐efficacy (*n* = 4), perception (*n* = 3), and satisfaction with learning and work (*n* = 5). Qualitative data revealed that participants appreciated the training opportunities, found the structured approach beneficial and suggested it for future clinical practice, were mindful of others’ roles and identities, and demonstrated awareness of patient safety. Several studies presented statistically significant results in their findings, while others did not.

**Table 4:** Outcomes measured post training.

Studies	Quantitative outcomes									Qualitative outcomes
	Communication	Completeness and documentation	Attitudes towards patients’ safety	Competence	Knowledge	Decreased in handover:	Confidence	Perception	Satisfaction in:	
1. Acharya et al., 2017	√		√				√^∗^		Learning	1. Educational opportunities; 2. Structured approach; 3. Aware of others’ roles.

2. Adam et al., 2022	√	√	√					√		

3. Beament et al., 2018					√		√			1. Challenges concerning patient factors and change management processes; 2. Practice enhancement, patient‐centered care, professional practice; 3. Grassroots initiatives and systems.

4. Cunningham et al., 2021	√									1. Clear communication to maintain patient safety; 2. Efficiently conveying the patient’s background

5. Fahajan et al., 2023	√^∗^		√^∗^					Using ISBAR^∗^	Work^∗^	

6. García Roig et al., 2020		√				Interruption^∗^				

7. Haddeland et al., 2022										1. Predictability and security; 2. Usability; 3. Recommendations for further use.

8. Holt et al., 2020				√^∗^			√^∗^			

9. Lebensohn‐Chialvo et al., 2022									Learning	

10. Lim & Kang, 2024				√			√		Learning	

11. Reilly et al., 2024	√^∗^									

12. Ronnebaum et al, 2023	√^∗^									

13. Soares et al., 2023		√								

14. Stuart et al, 2021					√					

15. Usher et al, 2018		√				Time^∗^		√^∗^		

16. Vanderzwan et al, 2023		√								

17. Wollenhaup et al, 2017		√							Work	

^∗^Statistical significance, *p* < 0.05.

#### 3.3.3. Theme 3: Inadequacy in Interprofessional Training on Clinical Handovers

Only four studies incorporated interprofessional training [24, 26, 35, 36]. The training programs involved lectures [24, 26, 36], videos [24, 35], debrief sessions [26, 35], simulations [36], and standardized patients [26, 35]. All four studies implemented SBAR or its derivatives in their training programs. Only one study involved more than two healthcare professionals [24]. Notably, Cunningham et al. [26] engaged doctors and allied health professionals who underwent training in TeamSTEPPS and SBAR. Subsequently, they assessed their communication competence using I‐PASS. In contrast, Beament et al. [24] included doctors, nurses, and allied health professionals but solely utilized iSoBAR in the training and measured their knowledge and confidence levels. Lebensohn‐Chialvo et al. [35] utilized a simulation feedback form to evaluate participants’ learning satisfaction, while faculty members employed an observation checklist to evaluate participants’ performance in patient assessment and care. It appears that the focus was not specifically on clinical handover within teams. In a qualitative study by Haddeland et al. [36], doctors and nurses who utilized ISBAR in communication reported increased awareness of communication dynamics and professional roles. While they acknowledged its appropriateness and potential for further utilization, there was a noted need for enhanced training. However, some doctors expressed disinterest in adopting the ISBAR tool due to feelings of being degraded.

Although communication is crucial and clinical handovers occur not only within single professions but also across interprofessional settings, there was a deficiency in standardized training for interprofessional to enhance communication during clinical handovers. The overarching objective of handover is improving patient safety yet none of the papers included in this review have this as a measured outcome.

## 4. Discussion

This scoping review mapped available literature on education strategies to promote clinical handover competence in clinical settings. We documented clinical handover competence by analyzing training methods, communication frameworks were commonly used in the training programs, measurement tools in clinical handover training, the adequacy in interprofessional training on clinical handovers and key findings on education strategies to promote clinical handover competence in clinical settings. Below, we provide important information on the implications of the findings and the gaps that emerged from the results of this review that can be relevant for healthcare professionals, educators, and researchers.

The reviews’ findings indicated that an effective training program should encompass various activities, starting with fundamental information on clinical handovers and communication delivered through lectures. Videos were deemed necessary to illustrate both effective and ineffective handover scenarios. Group discussions and debriefing sessions were found to be valuable in fostering reflection and reinforcing knowledge and skills. Simulations, including the utilization of simulators, role‐playing, standardized patients, and virtual simulations, were frequently employed to practice handovers. Following the COVID‐19 pandemic, online education became another widely accepted practice. In the cited studies, lectures [28, 33], debriefing sessions [31], and simulations [33] could be conducted online. However, Ronnebaum et al. [31] found that face‐to‐face debriefing sessions led to greater improvement in communication and content skills compared to online computerized personal reflection debriefing. For training in communication and clinical handover, traditional face‐to‐face learning is still beneficial. Basic principles of communication and clinical handovers can be effectively taught through online platforms, whereas interactive exercises such as hands‐on practice of handovers are best conducted in person. Studies indicate that experiential learning methods like simulations with standardized patients, which replicate authentic clinical environments and interactions, lead to enhanced learning satisfaction compared to traditional methods [35]. The findings suggest that a curriculum‐based program incorporating didactic to provide basic theory, experiential to hands‐on practice, and interactive learning approaches to engage healthcare professionals’ collaboration is more comprehensive and should be considered for healthcare professional training.

Research has demonstrated that simulated handover experiences, checklists, and mnemonics are effective in reducing errors [37]. Frameworks offer a systematic approach to conducting clinical handovers, enhancing the completeness of information shared verbally and in writing. SBAR and its derivatives are widely used and adaptable, making them suitable for adoption by diverse healthcare professionals. The use of an SBAR sheet [23] and I‐PASS form [21] has been shown to improve doctors’ written handover. Implementing a standardized form for verbal handovers and as written documentation has been shown to decrease errors related to missing information communicated to other healthcare team members. This standard format can be advocated for adoption by doctors and nurses in clinical practice. Usher et al. [28] provided nurses with a pocket card as a reminder of the critical nature of precise handovers in clinical settings. This intervention led to notable enhancements in perceptions of communication quality, handover accuracy and completeness, as well as a reduction in handover duration. The concept of pocket cards can be replaced with alternatives such as distributing printouts to be placed on medical record covers, displaying them on walls, or setting up computer pop‐ups could potentially yield similar effects.

The outcomes assessed following clinical handover training are varied. Successful clinical handover hinges on efficient communication, a key factor in ensuring patient safety. A majority of the studies reviewed highlighted substantial enhancements in communication. Additionally, confidence, another crucial attribute influencing effective clinical handovers, was also evaluated in the reviewed studies, with significant improvements noted. Interruptions by others during clinical handovers can disrupt the transmission of information and are associated with errors. García Roig et al. [21] and Soares et al. [33] proposed doctors to use the I‐PASS for handoffs and showed results of a significant improvement in reporting the key information and reducing interruptions in handoffs. From the viewpoint of physicians, it is crucial not only to convey accurate information effectively but also to minimize interruptions. In contrast, within other healthcare fields like nursing, studies have not assessed whether the communication framework will decrease interruptions indicating the need for additional research in this area. However, Usher et al. [28] involved nurses in their study to demonstrate that employing a communication framework enhanced the completeness of information and decreased handover duration. The studies examined primarily concentrated on training methods for single‐patient handovers. Given that nurses oversee many patients during a single shift, the duration of handovers is a critical issue. While prior discussions emphasized a decrease in interruptions primarily from physicians, nurses likewise must limit disruptions during handovers, particularly during shift changes, to mitigate errors and shorten handover periods. Likely as a result of this, the studies reviewed also assessed participants’ satisfaction with the training approach and its positive effect on job satisfaction. Within these 17 studies, 5 specifically evaluated participants’ satisfaction levels regarding the training methods and highlighted their influence on work efficiency and educational outcomes.

The overarching objective of handover is improving patient safety. The reviewed studies utilized various systems to enhance communication and assessed participants’ proficiency in clinical handovers. The interventions yielded significant outcomes, evident in enhanced confidence [12, 25], improved attitudes toward patient safety [30], and enhanced accuracy in documentation [21, 23, 28, 29, 33, 34], ultimately contributing to safer patient practices. The execution and assessment of initiatives with the potential to yield positive impacts on patient outcomes can be challenging within healthcare settings. The limited objective research on the overall reduction of healthcare risks associated with effective interprofessional and intraprofessional handovers is noted. The sustainability and application of ISBAR across diverse healthcare environments, particularly concerning deteriorating patients, warrant future evaluation [24].

Clinical handovers typically occur within the same healthcare professional group as well as involve different professionals, like nurses reporting patients’ deteriorating conditions to doctors, and physiotherapists handover patients to nurses. The studies included in the analysis indicated that staff confidence significantly increased post‐training, particularly when utilizing a structured handover tool [12, 24, 25, 32]. The staff’s enhanced confidence in communication and clinical handover leads to accurately conveying information and promoting patient safety. Communication must be standardized to be understandable and be used by the interprofessional team [38]. Therefore, the development of interprofessional education on clinical handovers that involves multiple disciplines and promoting the use of standardized format is crucial for inclusion in training programs. The studies lacked substantial information on interprofessional education concerning clinical handovers. Out of the four studies that involved interprofessional teams [24, 26, 35, 36], only one of them included more than two professions [24]. Even though all studies reported improvement in communication, knowledge, confidence, and satisfaction with learning, the outcomes did not reach statistical significance. Insufficient interprofessional training is a pressing necessity, particularly when various healthcare teams need to relay patients’ information to professionals in other disciplines.

In view of this, the incorporation of team‐based learning (TBL) is recommended for interprofessional training, a method extensively utilized in healthcare education. TBL involves an instructional strategy where students engage in small group learning, emphasizing a student‐centered and active learning approach. This method also integrates a “flipped classroom” model, where students come to class ready to collaborate in teams to address clinical problems, promoting learning within and among all teams. TBL is progressively adopted as an interprofessional educational strategy to enhance knowledge exchange and promote collaboration among various disciplines [39]. TBL can be designed starting with a preclass online lecture and a clinical handover demonstration video. This can be followed by a simulation training experience, small group discussions, and debriefing sessions. Utilizing a TBL approach with case scenarios that involve clinical handovers will additionally boost interaction and communication among students from various disciplines. Haddeland et al. [36] found that the use of ISBAR enhanced understanding of communication dynamics and professional roles. Given that the four studies incorporated interprofessional training [24, 26, 35, 36] utilized SBAR and its derivatives effectively convey essential information across different professions, healthcare education institutions should consider implementing SBAR into training program at an earlier stage as a universal tool to engage students in interprofessional education.

### 4.1. Limitations

One limitation of this study was the exclusion of non‐English studies, which could potentially affect the generalizability of the findings by overlooking diverse perspectives from other linguistic backgrounds. Including studies within the last 10 years may risk missing the key seminal research on handovers. The predominance of quantitative studies in the search results suggests that the depth and richness of healthcare professionals’ experiences with training programs aimed at improving clinical handovers might have been constrained. Consequently, the applicability of the findings of this scoping review may be restricted. Another limitation arises from the varying qualities of the 17 studies included, potentially impacting the overall reliability and generalizability of the findings. The diverse array of training methods, communication frameworks, and measurement tools employed across these studies might pose challenges in making direct comparisons or generalizing the results. Furthermore, some studies lacked detailed information on the training components. Lastly, the literature search did not include studies involving paramedics, who play a crucial role in transferring emergency patient care to other healthcare professionals. Future research on this topic should specifically consider including paramedics in the study population.

### 4.2. Study Implications

Insufficient evidence exists to demonstrate a standardized assessment tool for evaluating the efficacy of training programs aimed at enhancing clinical handovers. The adoption of a standardized checklist, such as utilizing SBAR, could offer advantages in delivering consistent and routine evaluations of students’ performance and the effectiveness of training initiatives. Future research should evaluate interprofessional education in clinical handover and consider assessing the effectiveness of a standardized SBAR evaluation checklist. Given that only a few out of the 17 studies involved interprofessional education, there is a clear need for research and implementation of interprofessional education strategies in clinical handover training programs. Healthcare faculties should consider standardizing curriculum‐based training programs including TBL utilizing a SBAR communication framework, comprising online didactic instruction to impart fundamental knowledge on clinical handover, experiential learning through simulations, interactive student discussions, and interdisciplinary collaboration. The integration of advanced technologies such as immersive virtual reality training, which provides dynamic ward environments and diverse patient conditions, promises to significantly enhance and enrich the training experience for future programs among different disciplines. Notably, the reviewed studies have yet to explore this method, highlighting its untapped potential for improving training outcomes. Additionally, there is a need for more randomized controlled trials and longitudinal studies to assess the long‐term effectiveness of these education strategies on improving clinical handover competence.

## 5. Conclusion

In summary, this scoping review analyzed a diverse range of training methods employed to enhance communication in handovers across different healthcare professionals. Communication framework was essential to provide a structured approach to enhance accurate and completeness of information. There is a need to develop standardized and comprehensive training programs utilizing SBAR as a universal tool to improve both verbal and written handovers in interprofessional education. This approach would enhance consistent and accurate interdisciplinary communication among healthcare professionals from different backgrounds. There is a pressing need for innovative training that leverages technology to create authentic real‐world scenarios for interprofessional education. Such initiatives are crucial in enhancing patient safety, promoting effective care coordination, and elevating the overall quality of healthcare services.

## Conflicts of Interest

The authors declare no conflicts of interest.

## Funding

This research was supported by Tung Wah College, Hong Kong, China.

## Supporting Information

We reported our study according to the Preferred Reporting Items for Systematic reviews and Meta‐Analyses extension for Scoping Reviews (PRISMA‐ScR) guidelines (File 1).

## Supporting information


**Supporting Information** Additional supporting information can be found online in the Supporting Information section.

## Data Availability

The data that support the findings of this study are available upon request from the corresponding author. The data are not publicly available due to privacy or ethical restrictions.
